# Identification of core genes in prefrontal cortex and hippocampus of Alzheimer's disease based on mRNA‐miRNA network

**DOI:** 10.1111/jcmm.17593

**Published:** 2022-11-19

**Authors:** Zhi‐Hang Huang, Hai Wang, Dong‐Mei Wang, Xiu‐Yun Zhao, Wen‐Wen Liu, Xin Zhong, Dong‐Mei He, Ben‐Rong Mu, Mei‐Hong Lu

**Affiliations:** ^1^ Chongqing Key Laboratory of Sichuan‐Chongqing Co‐construction for Diagnosis and Treatment of Infectious Diseases Integrated Traditional Chinese and Western Medicine, College of Medical Technology Chengdu University of Traditional Chinese Medicine Chengdu China; ^2^ School of Pharmacy Chengdu University of Traditional Chinese Medicine Chengdu China; ^3^ School of Basic Medical Sciences Chengdu University of Traditional Chinese Medicine Chengdu China; ^4^ Jiangsu Key Laboratory of Neuropsychiatric Diseases, Institute of Neuroscience Soochow University Suzhou China

**Keywords:** Alzheimer's disease, bioinformatics, hub genes, miRNA, mRNA

## Abstract

Alzheimer's disease (AD) is a neurodegenerative disorder with cognitive impairment and abnormal mental behaviour. There is currently no effective cure. The development of early diagnostic markers and the mining of potential therapeutic targets are one of the important strategies. This study aimed to explore potential biomarkers or therapeutic targets related to AD in the hippocampus and prefrontal cortex, two brain regions highly related to AD. Differentially expressed genes and miRNAs between AD patients and healthy controls were obtained from the Gene Expression Omnibus database. The mRNA‐miRNA network was constructed and key genes involved in AD were screened out by protein–protein interaction analysis, and were subsequently verified by independent datasets and qPCR in an AD mouse model. Our findings showed that six hub genes including CALN1, TRPM7, ATR, SOCS3, MOB3A and OGDH were believed to be involved in the pathogenesis of AD. Western blot analysis further determined that CALN1, ATR and OGDH were the possible biomarkers and therapeutic targets for AD. In addition, 6 possible miRNAs biomarkers have also been verified by qPCR on AD animal models. Our findings may benefit clinical diagnosis and early prevention of AD.

## 1 INTRODUCTION

Alzheimer's disease (AD) is the main cause of dementia and has become one of the world's greatest disease crises.^1^ The number of people suffering from AD continues to increase. Senile plaques aggregated by amyloid‐β and neurofibrillary tangles formed by highly phosphorylated tau protein are the two main pathological features. Evidence indicates that genomic, cerebrovascular, metabolic and environmental factors may be related to AD.^2^, ^3^ However, the pathogenesis of AD is extremely complex and largely unknown. Notably, current drugs are only symptomatic treatment without halting the disease progression.^4^, ^5^ Therefore, exploration of the risk factors remains crucial, which helps in the early diagnosis and follow‐up intervention.

Nowadays, the importance of gene silencing induced by microRNAs (miRNAs) in regulating gene expression is evident.^6^, ^7^ The miRNAs bind to the 3′‐untranslated region of the mRNA to degrade or/and repress the translation of target genes.^8^ Interestingly, each miRNA may target hundreds of mRNAs simultaneously with a tightly regulated pattern. The regulatory role of miRNA‐mRNA network is involved in the most biological processes. Therefore, it has become a very important strategy to investigate the underlying mechanisms of human diseases including cancer,^9^ cardiovascular diseases^10^ and neurodegenerative disease.^11^, ^12^

At present, growing evidence has demonstrated that the deregulated expression of miRNAs is highly associated with the pathogenesis of AD.^13^, ^14^, ^15^, ^16^ They may exert impacts on regulating the key genes involved in AD such as amyloid precursor protein^17^ and beta‐secretase enzyme,^18^ the neuronal function and synapse plasticity,^19^ autophagy^20^ and signalling pathway.^21^, ^22^ Moreover, some miRNAs are highly linked to age and multiple cognitive functions, suggesting their potential utilization for diagnosis of AD and AD‐related cognitive dysfunction.^23^ However, screening key genes and miRNAs involved in AD based on the bioinformatics analysis of miRNA‐mRNA network analysis are still relatively lacking.

Considering alterations in the hippocampus and prefrontal cortex have been established to be associated with AD, this study is committed to explore key molecules in these two regions. Datasets of miRNAs and mRNAs from AD patients and healthy human were selected from GEO. The hub genes were screened out by constructing the mRNA and miRNA networks and further verified through independent datasets based on bioinformatics method and simultaneously verified by qPCR in an AD mouse model. These findings reveal new potential diagnostic and therapeutic biomarkers in AD therapy and provide novel insights on unravelling the pathogenesis of AD.

## 2 METHODS

### 2.1 Alzheimer's disease datasets

The expression datasets of six microarrays including GSE33000, GSE48552, GSE36980, GSE28146, GSE147232 and GSE159699^24^, ^25^, ^26^, ^27^, ^28^, ^29^ were obtained from the Gene Expression Omnibus (GEO) database (https://www.ncbi.nlm.nih.gov/geo/). The samples of three databases of GSE33000, GSE48552 and GSE36980 are all from the prefrontal cortex. GSE33000 dataset included 157 normal controls and 310 AD samples. GSE48552 dataset that included six healthy humans and six AD patients was analysed by high‐throughput sequencing. GSE36980 dataset (18 normal controls and 15 AD samples) was applied as an independent dataset for verification.

The datasets of GSE28146 and GSE159699 were from hippocampus, while GSE147232 was from plasma. GSE28146 dataset included 8 healthy controls and 22 AD patients. In the GSE147232 dataset, 5 normal and 5 AD patients were enrolled in the study. The expression profile of GSE159699 was analysed by high‐throughput sequencing as a validation dataset which included 12 AD patients and 18 healthy humans.

### 2.2 Animals

The APP/PS1 mice model of AD on a C57BL/6, C3H genetic background were used in this study. It is a commonly used mouse model for AD research. They contain human Swedish APP and Presenilin‐1 (PS1) mutants. Amyloid‐β appears in the brain at 3 months, and a large amount of it deposits in the hippocampus and cortex at 6–7 months when the animals display severe neuroinflammation, synaptic loss and cognitive deficiency. Five transgenic mice aged 7–8 months and age and gender‐matched wild‐type mice (two pairs of males and one pair of females) were used for qPCR and western blot analysis. The transgenic mice were purchased from Jackson Laboratory (stock number 004462) and housed under SPF condition. The animal study was reviewed and approved by Ethics Committee of University Chengdu TCM.

### 2.3 Differential expression analysis

The GEO2R, a free web tool (https://www.ncbi.nlm.nih.gov/geo/geo2r/), or edgeR package of R (version 3.6.2), an R‐based tool within the Bioconductor project was used to identify the DEGs or DEMs in two or more groups of samples in the GEO series. Herein, GSE33000, GSE36980, GSE28146 and GSE147232 were analysed by GEO2R to obtain DEGs or DEMs between normal and AD samples. GSE48552 and GSE159699 were analysed by edgeR package of R. In these datasets, DEGs or DEMs with |log2 Fold Change| ≥ 0.2 and *p* value < 0.05 were considered as significantly different for GSE33000 and GSE36980, and |log2 Fold Change| ≥ 1 and *p* value < 0.05 were considered as significantly different for other datasets. The corresponding gene of the probe is blank or the repeated value is deleted. Finally, the volcano map or heatmap in the ggplot2 package of R were used to show DEGs and DEMs.

### 2.4 Selection of DEMs and their target genes

After screening the eligible miRNAs by the above methods, the top 15 up‐regulated and down‐regulated miRNAs from GSE48552 and the top 10 up‐regulated and down‐regulated miRNAs from GSE147232 were selected for further analysis. The selected up‐regulated and down‐regulated miRNAs were then analysed by the miRNET website (https://www.mirnet.ca/) to obtain the target genes.^30^ The miRNET is a relatively powerful and comprehensive miRNAs target gene prediction tool which integrates data from 14 different miRNA databases.

### 2.5 GO and KEGG enrichment analysis

DAVID 6.8 (https://david.ncifcrf.gov/tools.jsp) provides researchers with a comprehensive set of functional annotation tools to understand the biological significance behind a large number of genes. Here, it was used for Gene Ontology (GO) and Kyoto Encyclopedia of Genes and Genomes (KEGG) analysis to obtain the target enrichments and the signal pathways of DEGs and DEMs. GO is a widely used ontology in the field of bioinformatics,^31^ which covers three aspects of biology: biological process (BP), molecular function (MF), and cell component (CC). KEGG has a powerful graphical function to introduce the numerous metabolic pathways and the relationships of the pathways.^32^ These enrichments and pathways were used to draw the bubble chart in the ggplot2 package of R and *p* < 0.05 is taken as the critical value.

### 2.6 The network construction of mRNA‐miRNA and hub genes identification

Networks of DEG and DEMs from the prefrontal cortex, and DEGs from the hippocampus and DEMs from the plasma were constructed networks, respectively. The common genes of up‐regulated DEGs and down‐regulated DEMs target genes, as well as down‐regulated DEGs and up‐regulated DEMs target genes were selected. Venn diagrams (http://bioinformatics.psb.ugent.be/webtools/Venn/) were used to find common genes between DEGs and DEMs.^33^ The cytoHubba plugin in Cytoscape are usually used to explore important nodes / hubs and fragile patterns in interactive group networks by several topology algorithms, such as degree, edge penetration, maximum neighbourhood component (MNC) and Maximal Clique Centrality (MCC). It was used here to screen hub genes between DEGs and DEMs targets. According to the results of the MCC, the top genes were selected as the candidate hub genes.

### 2.7 Validation of hub genes by a microarray

GSE36980 and GSE159699 expression profiles were used to verify hub genes in prefrontal cortex and hippocampus, respectively. The *t*‐test was used to compare the expression of these genes between normal and diseased groups. The ggpubr package in R language was used to show the expression comparison between genes, and *p* < 0.05 was considered as the significant gene.

### 2.8 Quantitative PCR analysis

Quantitative PCR were also performed to examine the level of mRNA of hub genes and related miRNAs. Mice were anaesthetised and the blood samples were obtained from the orbital venous plexus. Then, they were perfused with saline and decapitated. The total RNAs of brain and plasma were extracted by Trizol regent (Thermo Scientific, Rockford, IL, USA) following the manufacturer's instructions. The first‐strand was generated using the First Strand cDNA Synthesis kit (Thermo Scientific, Rockford, IL, USA) and Mir‐X miRNA First‐Strand Synthesis (Takara, Dalian, China), respectively. Gene‐specific primers used are listed in Table S1. RT‐PCR was accomplished using the FastStart Universal SYBR Green Master (Rox) (Roche) in the ABI PRISM® 7500 real‐time PCR system (Applied Biosystems, Foster, CA, USA). GADPH and U6 were used as endogenous controls, respectively. Relative expression level was computed using 2^−ΔΔCt^ method.

### 2.9 Western blot analysis

Protein of brain tissue were homogenized with brain lysis buffer (50 mm Tris HCl, pH 7.5, 5 mm EDTA, 1% Triton X‐100) with protease inhibitor (Roche Diagnostics). The samples were boiled for 5 min and were separated by sodium dodecyl sulphate polyacrylamide gel electrophoresis (SDS‐PAGE). After transferring to polyvinylidene difluoride membrane (Millipore, Amsterdam‐Zuidoost, The Netherlands, IPFL00010), the membranes were blocked with 5% non‐fat milk for 1 h at room temperature and probed overnight at 4°C with primary antibodies against CALN1, TRPM7, ATR, SOCS3, MOB3A and OGDH (TRPM7 were purchased from Abcam, the others were from Proteintech). Membranes were then washed, incubated with appropriate peroxidase‐conjugated secondary antibodies and developed by ECL (GE Bioscience) to detect protein signals.

### 2.10 Statistical analysis

The data of gene expression by PCR and protein levels assessed by western blot are represented as mean ± SD and analysed by SPSS 17.0. Student's t‐test was used for two group comparisons. Statistical analysis of bioinformatics is carried out through its own software. *p < 0.05; **p < 0.01; ***p < 0.001.

## 3 RESULTS

### 3.1 Identification of DEGs and DEMs

**FIGURE 1:**
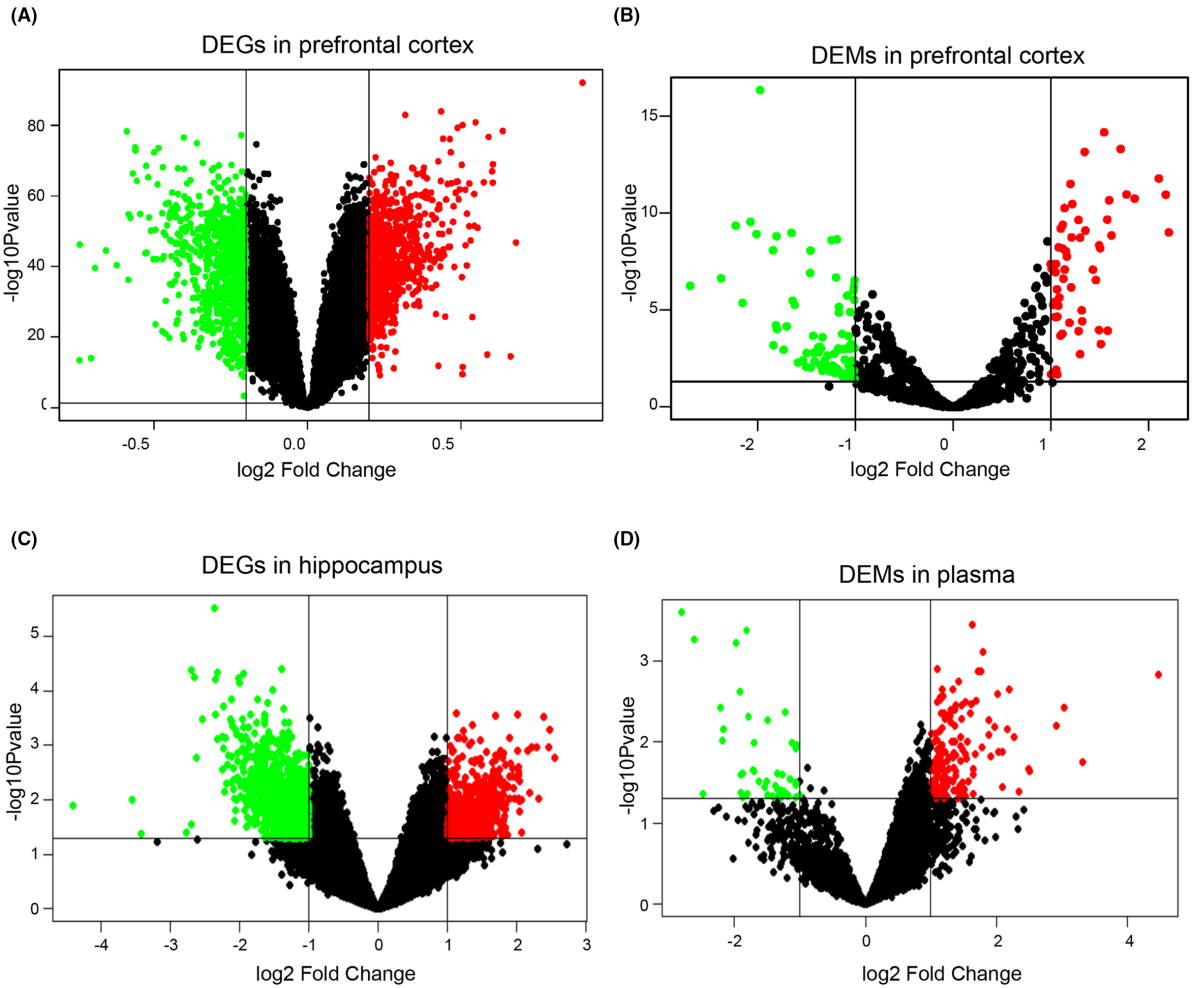
Identification of DEGs and DEMs. (A and C) DEGs in prefrontal cortex (A) and hippocampus (C). (B and D) The DEMs in prefrontal cortex (B) and hippocampus (D). DEGs or DEMs with |log2 Fold Change| ≥ 0.2 (A) or 1 (B–D) and *p* value < 0.05 were considered as significantly different. Red and green represent up‐regulated and down‐regulated expression, respectively

For the datasets of prefrontal cortex, there was a total of 1595 DEGs which included 834 up‐regulated and 761 down‐regulated genes in GSE33000 dataset according to GEO2R analysis (Figure 1A). The edgeR package of R analysis showed that in GSE48552 there was a total of 139 DEMs which included 56 up‐regulated and 83 down‐regulated miRNAs (Figure 1B).

According to the method mentioned above, we also analysed DEGs in hippocampus and DEMs in plasma. As a result, a total of 1500 DEGs including 573 up‐regulated and 927 down‐regulated genes from GSE28146 dataset, and a total of 120 DEMs including 88 up‐regulated and 32 down‐regulated DEMs were obtained from GSE147232 dataset, respectively (Figure 1C and D). All DEGs and DEMs were shown by volcano map.

### 3.2 Prediction of the target genes of DEMs

By pairing with mRNA bases of target genes, miRNA guided the silencing complex to degrade mRNA or block its translation. At present, miRNA plays an important role in cell differentiation, biological development, and the development of multiple diseases. Thus, analysing DEMs and the corresponding target genes can help explore the regulatory network of miRNA and mRNA, and find the key regulatory signalling pathways and molecules involved. The miRNET was used to predict the potential targets of miRNAs. For DEMs of prefrontal cortex, only 4 of the top 15 up‐regulated miRNAs, and 2 of the top 15 down‐regulated miRNAs have corresponding target genes. Therefore, these 4 up‐regulated miRNAs and 2 down‐ regulated DEMs were selected to screen their downstream target genes (Table 1). For DEMs from plasma, the top 10 up‐regulated and down‐regulated miRNAs were selected for subsequent target genes screening (Table 2).

**TABLE 1:** The top 4 up‐regulated and the top 2 down‐regulated miRNAs

miRNAs	up‐regulated			down‐regulated		
		LogFC	*p* value		LogFC	*p* value
1	I‐mir‐18a‐5p	1.579619	2.17E‐10	hsa‐miR‐212‐3p	−1.65184	1.06E‐09
2	hsa‐mir‐141‐3p	1.583741	1.19E‐04	hsa‐mir‐212‐5p	−1.80367	1.59E‐09
3	hsa‐miR‐99b‐5p	1.496446	4.63E‐09			
4	hsa‐mir‐153‐3p	1.715996	4.96E‐14			

**TABLE 2:** The top 10 up‐regulated and down‐regulated miRNAs

miRNAs	up‐regulated			down‐regulated		
		LogFC	*p* value		LogFC	*p* value
1	hsa‐miR‐590‐5p	4.480462	0.001474	hsa‐miR‐4722‐5p	−2.8073	0.000246
2	hsa‐miR‐3928‐3p	3.322292	0.017716	hsa‐miR‐3200‐3p	−2.61103	0.000534
3	hsa‐miR‐33a‐5p	3.037683	0.003767	hsa‐miR‐5009‐5p	−2.48688	0.043831
4	hsa‐miR‐744‐3p	2.498516	0.023013	hsa‐miR‐550b‐3p	−2.21349	0.003759
5	hsa‐miR‐4506	2.488517	0.021234	hsa‐miR‐4756‐3p	−2.18068	0.009546
6	hsa‐let‐7f‐5p	2.275484	0.008674	hsa‐miR‐550a‐3p	−2.17163	0.007008
7	hsa‐miR‐3118	2.187842	0.002202	hsa‐miR‐5194	−1.98317	0.000603
8	hsa‐miR‐4770	2.164066	0.006919	hsa‐miR‐3662	−1.91111	0.002395
9	hsa‐miR‐3155a	2.084135	0.013079	hsa‐miR‐3929	−1.90145	0.041515
10	hsa‐miR‐4698	2.015431	0.002566	hsa‐miR‐362‐3p	−1.87701	0.048337

**FIGURE 2:**
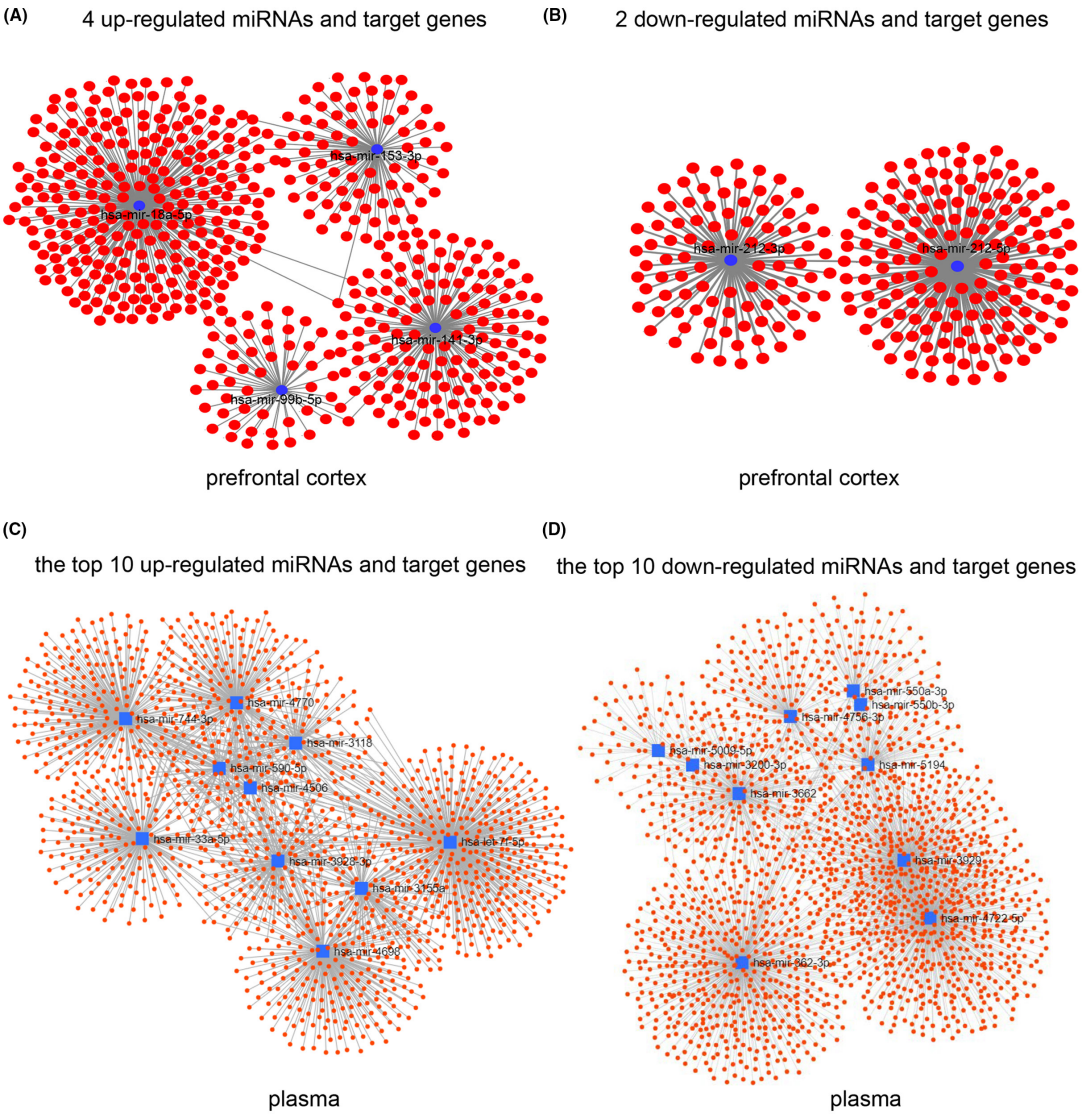
Analysis of the target genes of DEMs. (A and B) Network of the 4 up‐regulated miRNAs (A) and the 2 down‐regulated miRNAs (B) with their target genes in prefrontal cortex. (C and D) Network of the top 10 up‐regulated miRNAs (C) or the top 10 down‐regulated miRNAs (D) and their target genes

The miRNET analysis showed that 527 genes mediated by 4 up‐regulated miRNAs (Figure 2A), and 251 genes mediated by 2 down‐regulated miRNAs (Figure 2B) were obtained in prefrontal cortex, respectively. In plasma, 1838 genes mediated by the top 10 up‐regulated miRNAs (Figure 2C), and 1492 genes mediated by the top 10 down‐regulated miRNAs (Figure 2D) were obtained, respectively.

### 3.3 The functional enrichment analysis of DEGs

**FIGURE 3:**
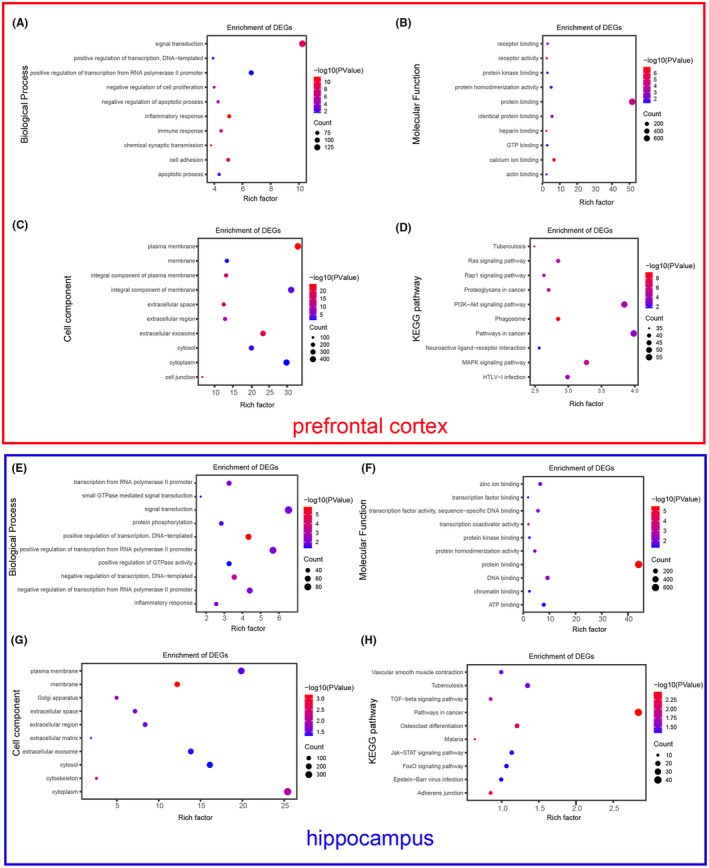
The functional enrichment analysis of DEGs. (A–C and E–G) GO enrichments analysis of DEGs at three aspects of biology: BP (A, E), MF (B, F) and CC (C, G) in prefrontal cortex and hippocampus as indicated in graph. (D and H) KEGG pathways enrichment analysis of DEGs in prefrontal cortex (D) and hippocampus (H). The size of the dot represents the number of genes, and the red represents the smaller p‐value, the blue represents the larger p‐value

To identify biological effects related to biological phenomena, GO and KEGG enrichments were used to analyse these DEGs. The DEGs were categorized into BP, MF and CC for GO enrichment analysis. For the prefrontal cortex, genes for BP were mainly enriched in signal transduction and inflammatory response (Figure 3A); genes for MF were mainly enriched in protein binding and calcium ion binding (Figure 3B); genes for CC were mainly enriched in plasma membrane and extracellular exosome (Figure 3C). In addition, KEGG pathways were significantly enriched pathways in cancer and phagosome (Figure 3D).

For hippocampus, genes for BP were mainly enriched in positive regulation of transcription, DNA‐templated/negative regulation of transcription, DNA‐templated/ negative regulation of transcription from RNA polymerase II promoter (Figure 3E); genes for MF were mainly enriched in protein binding, transcription coactivator activity, DNA binding (Figure 3F); genes for CC were mainly enriched in membrane, cytoskeleton, cytoplasm (Figure 3G). In addition, KEGG pathways were significantly enriched in cancer, tuberculosis and osteoclast differentiation (Figure 3H).

### 3.4 The functional enrichment analysis of DEMs target genes

**FIGURE 4:**
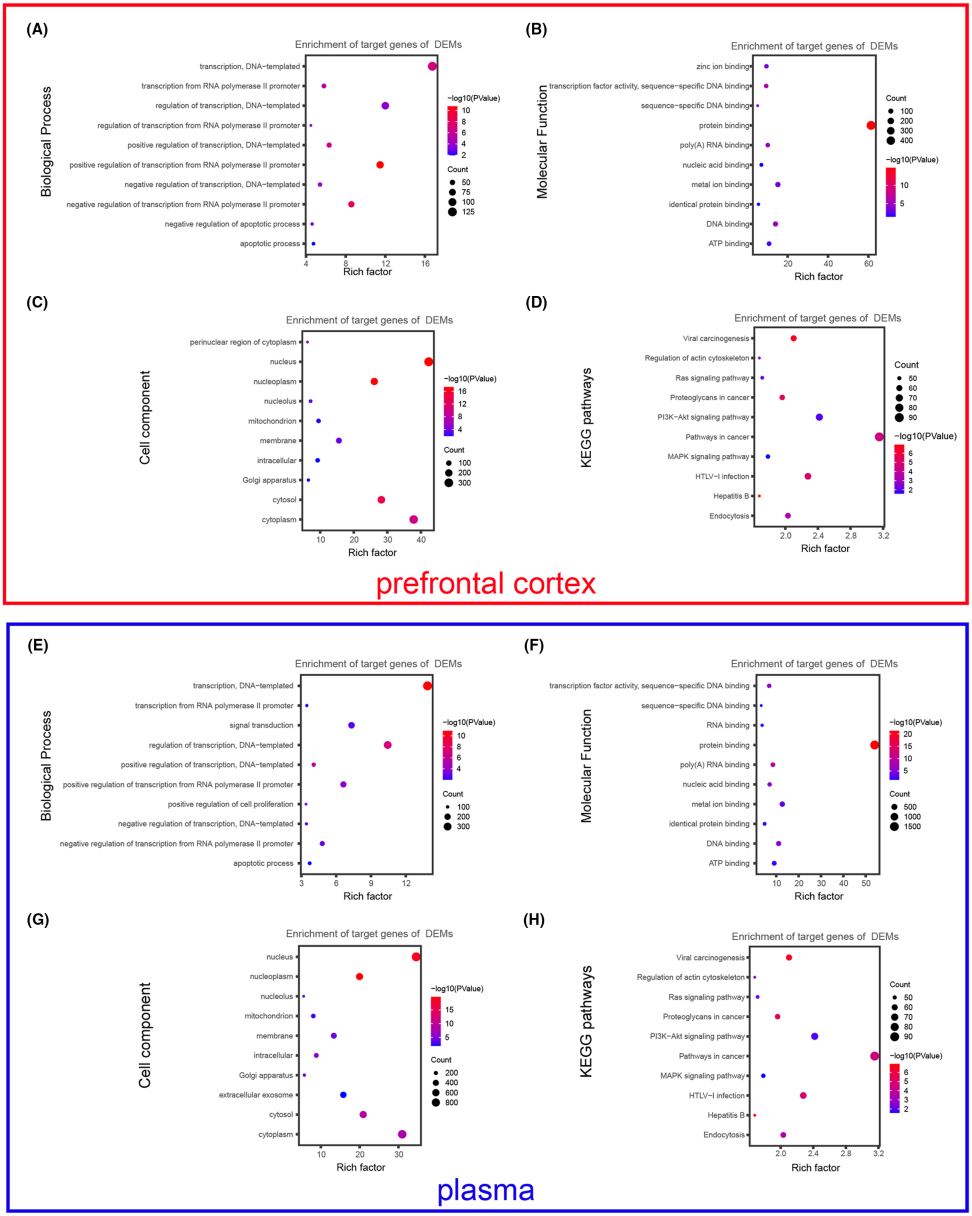
The functional enrichment analysis of DEM target genes. (A–C and E–G) GO enrichments analysis of DEMs target genes at three aspects of biology: BP (A, E), MF (B, F) and CC (C, G) in prefrontal cortex and plasma as indicated in graph. (D and H) KEGG pathways enrichment analysis of DEM target genes in prefrontal cortex (D) and plasma (H). The size of the dot represents the number of genes, and the red represents the smaller p‐value, the blue represents the larger p‐value.

The target genes of selected DEMs were also analysed by GO and KEGG enrichments, respectively. Results from the GO analysis indicated that, DEMs target genes of prefrontal cortex were mostly involved in positive and negative regulation of transcription from RNA polymerase II promoter at the level of BP (Figure 4A), while those of plasma were mostly involved in transcription/DNA‐templated and regulation of transcription/ DNA‐templated (Figure 4E). DEM target genes from both prefrontal cortex and plasma showed similar enrichments at the level of BP and MF. These targets genes in MF were both generally enriched in protein binding (Figure 4B and F). And, in CC they were mainly both focused on the nucleus and nucleoplasm (Figure 4C and G). In addition, KEGG pathways analysis indicated that the target genes were predominantly enriched in pathways in cell cycle in prefrontal cortex (Figure 4D), while in viral carcinogenesis and proteoglycans in cancer in plasma (Figure 4H).

### 3.5 mRNA‐miRNA network construction and hub genes identification

As known, miRNA was bound to the associated region of mRNA and terminated mRNA transcription or translation. Therefore, we tried to construct the mRNA‐miRNA network in prefrontal cortex and hippocampus to better understand the relationship of these molecules and identify the key regulators involved in AD. Unfortunately, no hippocampal miRNA dataset is available. In this study, miRNA dataset from plasma instead of hippocampus is used to construct the network. Venn Diagram was used to map the common genes between up‐regulated DEGs and down‐regulated DEM target genes, and the common genes between down‐regulated DEGs and up‐regulated DEM target genes.

**FIGURE 5:**
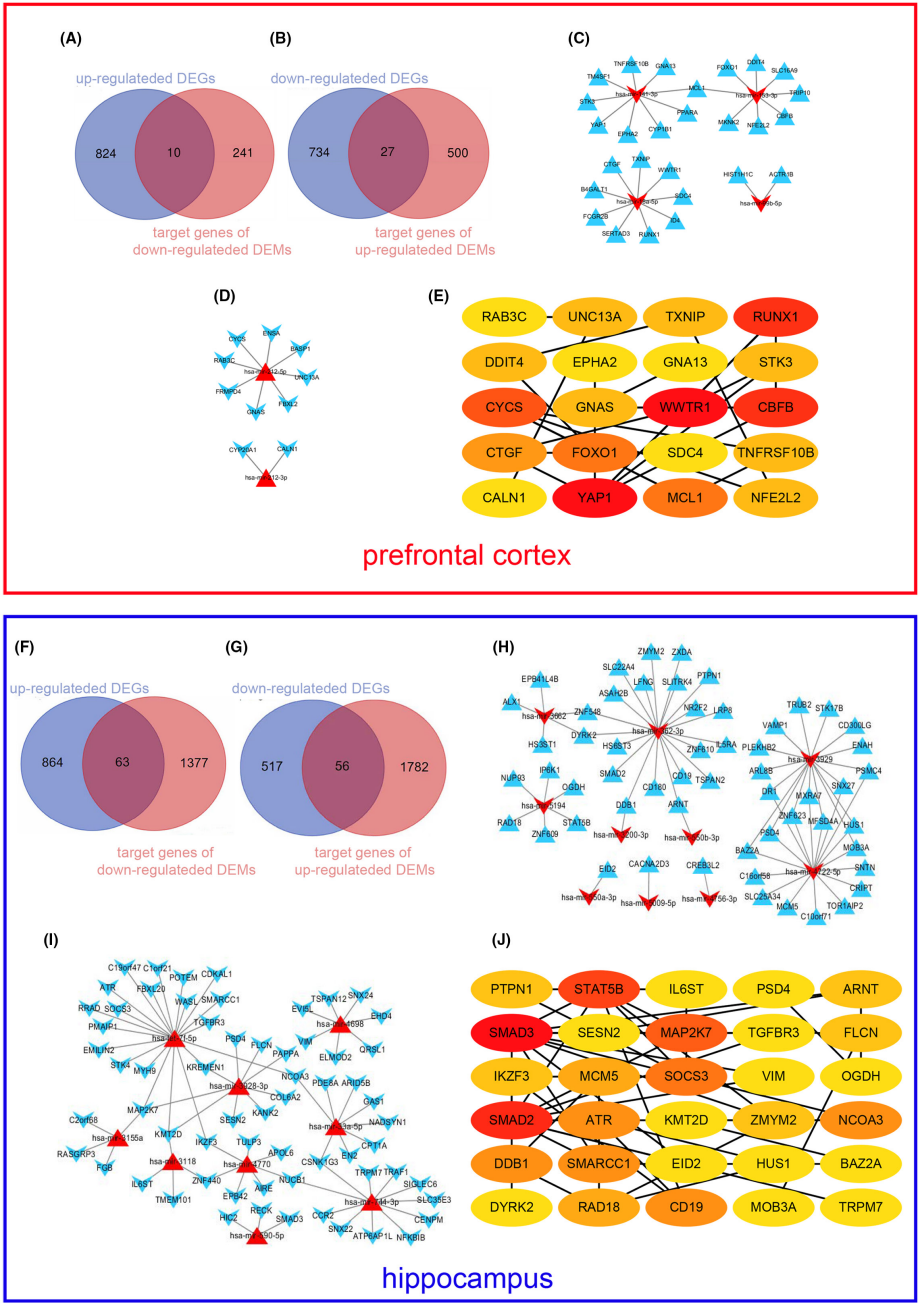
The mRNAs and miRNAs network. (A, B, F and G) The common genes between up‐regulated DEGs and down‐regulated DEM target genes (A and F) or between down‐regulated DEGs and up‐regulated DEM target genes (B and G) in prefrontal cortex and hippocampus as indicated in graph. (C, D, H and I) The network between up‐regulated common genes and their corresponding down‐regulated DEMs (C and H) or between down‐regulated common genes and their corresponding up‐regulated DEMs (D and I). (E and J) Hub genes in prefrontal cortI(E) and hippocampus (J) were identified by Cytohubba plug‐ins in Cytoscape. And the darker the nodes, the higher the score

In prefrontal cortex, the results showed that there were 10 common genes between 834 up‐regulated DEGs and 251 down‐regulated DEM target genes (Figure 5A), and 27 common genes between 761 down‐regulated DEGs and 527 up‐regulated DEM target genes, respectively (Figure 5B). Ten common genes with 2 corresponding down‐regulated miRNAs and 27 common genes with 4 corresponding up‐regulated miRNAs were used to construct the mRNA‐miRNA networks by Cytoscape (Figure 5C and D). Finally, the top 20 genes with high scores were screened as candidate hub genes through MCC analysis by Cytohubba plug‐ins in Cytoscape (Figure 5E). These genes were analysed by GO and KEGG pathways, but there was no significant KEGG pathway for these genes (Table S2).

Similarly, 56 common genes between 517 up‐regulated DEGs and 1782 target genes of down‐regulated DEMs (Figure 5F), and 63 common genes between 864 down‐regulated DEGs and 1377 target genes of up‐regulated DEMs were obtained in hippocampus and plasma (Figure 5G). Next, these 56 genes with 10 corresponding down‐regulated miRNAs and 63 genes with 9 corresponding up‐regulated miRNAs were screened. Furthermore, these 119 genes and 19 miRNAs were used to construct mRNA and miRNA networks by Cytoscape (Figure 5H and I). Among these common genes, the top 30 genes with the highest scores were determined as candidate hub genes through MCC as mentioned above (Figure 5J). Moreover, these candidate hub genes were analysed by GO enrichment and KEGG pathway, and the results were shown in Table S3.

### 3.6 Verification of hub genes

To determine the accuracy of above candidate hub genes, two independent datasets of GSE36980 (prefrontal cortex) and GSE159699 (hippocampus) were used to verify the expression levels of them in normal and AD samples. GEO2R analysis initially showed that there were 653 DEGs (499 up‐regulated and 154 down‐regulated) in prefrontal cortex, and 397 DEGs (195 up‐regulated and 202 down‐regulated) in hippocampus. All DEGs in these two datasets were shown by volcano map (Figure S1A and B) and heatmap (Figure S1C and D).

**FIGURE 6:**
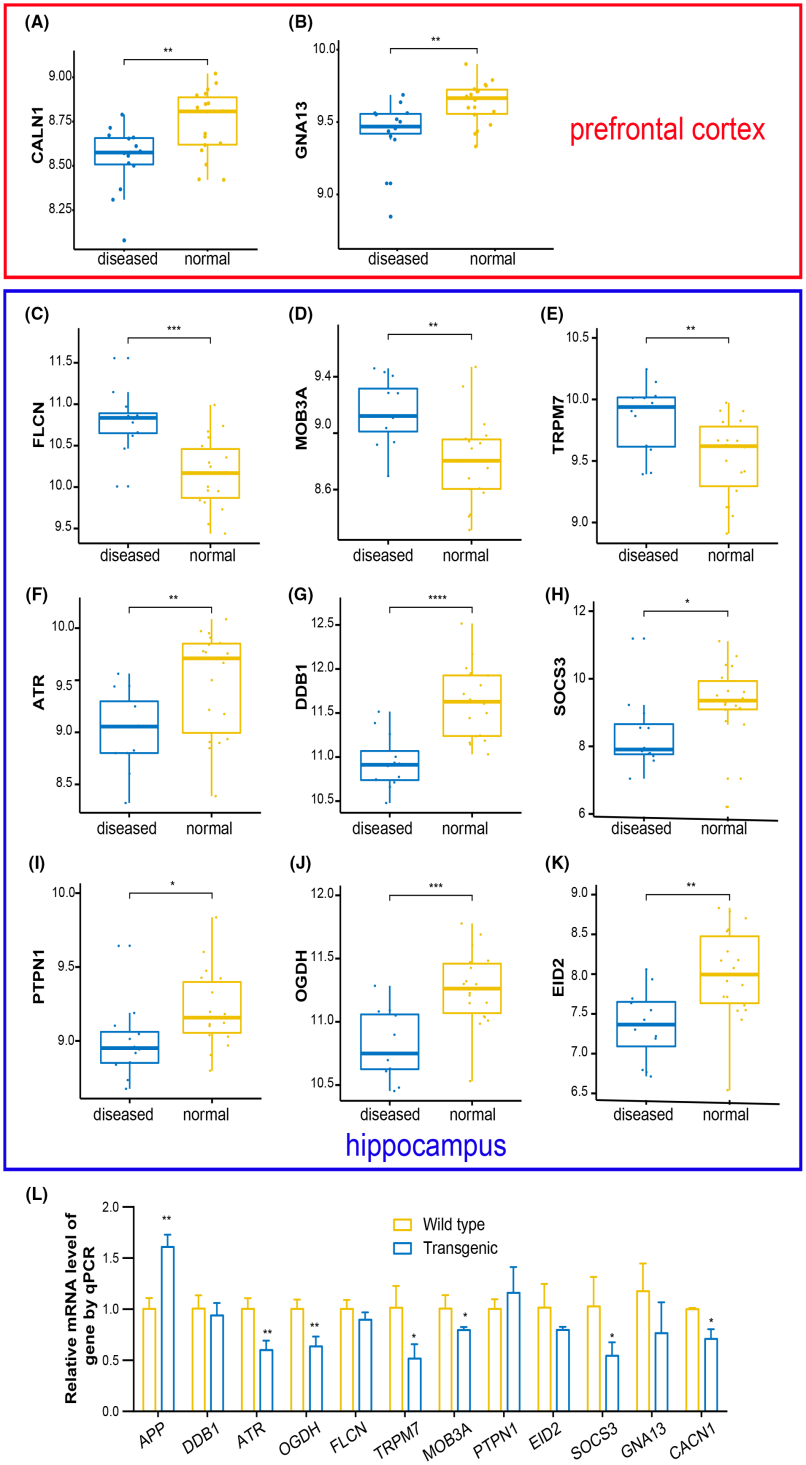
Verify the expression of hub genes in normal controls and diseased samples. The boxplots were used to show the expression level of these genes. (A) CALN1, (B) GAN13, (C) FLCN, (D) MOB3A, (E) TRPM7, (F) ATR, (G) DDB1, (H) SOCS3, (I) PTPN1, (J) OGDH, (K) EID2. (L) qPCR analysis in an APP/PS1 model mice of AD. Data are represented as mean ± SD. Student t‐test was used for two group comparisons. *p < 0.05, **p < 0.01, ***p < 0.001, ****p < 0.0001

Furthermore, the results of gene expression level showed that only two of the 20 candidate hub genes, CALN1 and GNA13, in prefrontal cortex were significantly decreased in AD samples (Figure 6A and B). And 9 of 30 candidate hub genes in hippocampus were remarkably altered. Three genes including FLCN, MOB3A and TRPM7 were significantly increased (Figure 6C–E), while the remaining six genes including ATR, DDB1, SOCS3, PTPN1, OGDH and EID2 were significantly decreased (Figure 6F–K) in AD samples compared with normal ones.

**FIGURE 7:**
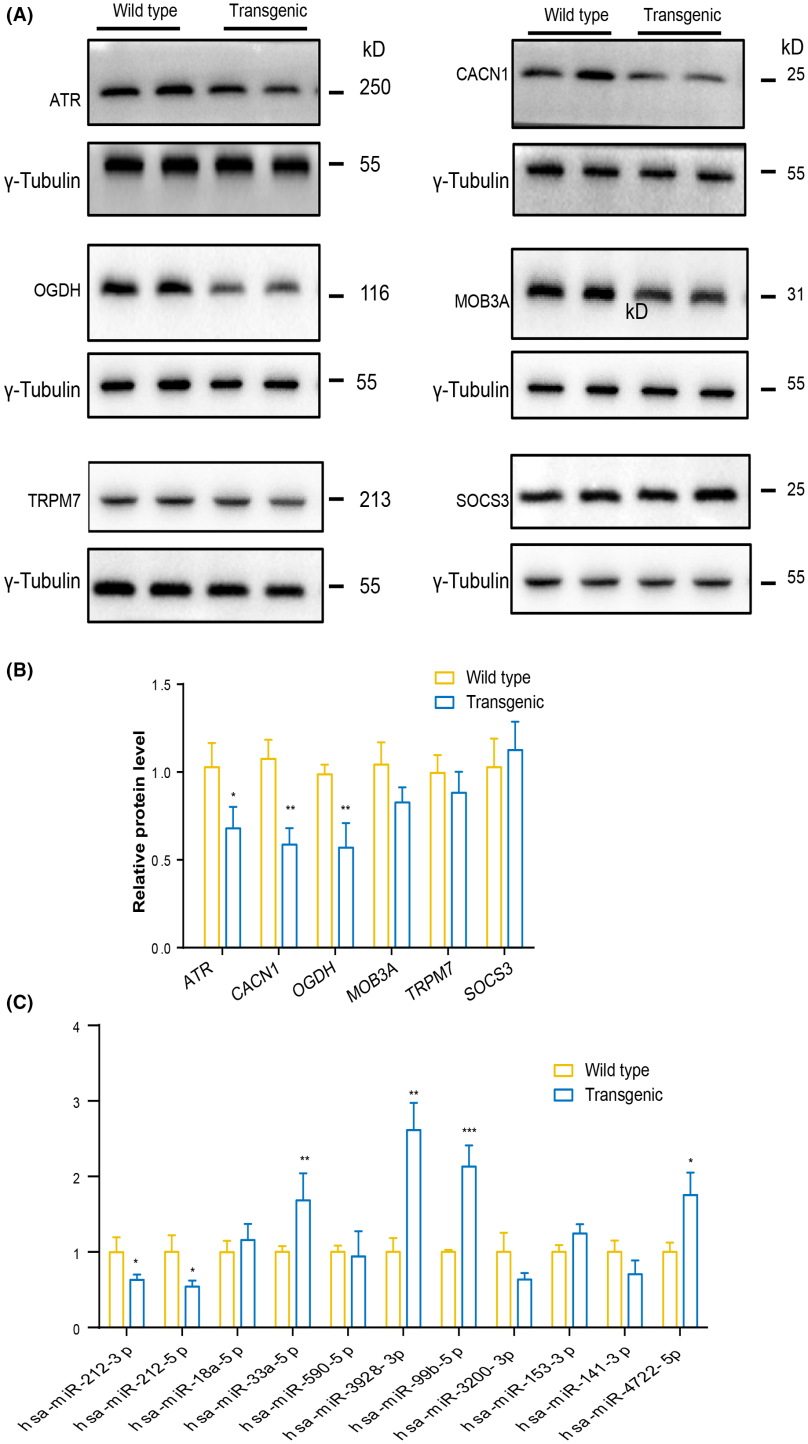
Verify the protein level of hub genes and potential miRNAs level in APP/PS1 mice. (A and B) Western blot analysis of candidate molecules involved in AD. (A) representative images of western blot. (B) the statistical analysis. (C) the qPCR analysis of key miRNAs. Data are represented as mean ± SD. Student t‐test was used for two group comparisons. *p < 0.05, **p < 0.01, ***p < 0.001.

Finally, to confirm whether the expression of these molecules is indeed affected in AD, we also carried out qPCR and western blot analysis on 7‐8‐month‐old APP/PS1 model mice of AD, which is very popular in AD research and exhibits obvious cognitive impairment at 7–8 months of age. The qPCR results show that among the 11 genes verified by datasets, 6 genes including CALN1, TRPM7, ATR, SOCS3, MOB3A and OGDH, are significantly changed. As expected, APP, as a positive control gene, the expression level in APP/PS1 mice was indeed significantly higher than that of wild type (Figure 6L). However, only CALN1, ATR and OGDH protein levels were significantly altered in AD mice models (Figure 7A and B). MOB3A shows a tendency of reduction, while there is no change in SOCS3 and MOB3A (Figure 7A and B). In addition, the top 11 miRNAs from prefrontal cortex and plasma were also tested by qPCR, and we found that six of them were significantly changed (Figure 7C).

## 4 DISCUSSION

AD is a chronic degenerative disease of the nervous system. And, it has become a major challenge for global health and has brought heavy burden on the economy and the society. Although it has been discovered long ago that amyloid plaques and neurofibrillary tangles are two hallmarks in AD, and significant progress in preclinical and clinical research was made in past decades,^34^ there is no effective cure so far. Considering the complex pathogenesis of AD, attentions paid to explore early diagnosis and effective intervention are still of priority. After decades of development, the core biomarkers in the cerebrospinal fluid of AD, including AB42, T‐tau and P‐tau, have entered clinical diagnostic criteria for AD.^35^ In fact, other biomarkers and risk factors related to AD have been gradually revealed. In addition to the well‐known APP and PS1 mutants in familial AD, APOE4 is currently considered to be the strongest risk gene for sporadic AD.^36^ Moreover, synaptic‐related proteins, such as neurogranin and synaptosomal‐associated protein 25,^37^, ^38^ mitochondrial function‐related Mitofusin‐1 and Mitofusin‐2,^39^ and glial cell‐specific TREM2^40^ can also offer information for the diagnosis of AD.

In recent years, as miRNAs have received more and more attention in AD,^14^, ^15^ the role of miRNA‐mRNA regulatory network in the pathology of AD would be of great interest. In this study, we analysed the important miRNA‐mRNA regulatory networks in prefrontal cortex and hippocampus. Six miRNAs and 37 mRNAs in prefrontal cortex, and 19 miRNAs and 119 mRNAs in hippocampus were obtained by constructing AD‐specific mRNA‐miRNA network. Finally, 6 hub genes including CALN1, MOB3A, TRPM7, ATR, SOCS3 and OGDH were identified and verified via another independent RNA‐seq datasets and qPCRs. Significantly, western blot indicated that CALN1, ATR and OGDH are possibly novel biomarkers for AD. Therefore, our findings may add new biomarkers for the diagnosis and provide new targets for the study on pathological mechanism of AD.

Although the protein level of TRPM7, SOCS3 MOB3A showed no significant changes in this study, we cannot rule out their possible role in AD. This may be restricted by the AD model we used here, the sample size, the animal age we tested and other factors. In fact, studies on TRPM7and SOCS3 have shown that they may be involved in the pathology of AD. TRPM7, as a divalent cation channel protein, contains an α‐ kinase domain at the intracellular C‐terminus. In the central nervous system, it is believed to be involved in synaptic and cognitive functions.^41^ TRPM7 maintain the normal function of presenilin, one component of γ‐secretase responsible for Aβ generation, through regulation of calcium entry in familiar AD‐related presenilin mutants.^42^ In addition, increasing cleavage of TRPM7 α‐kinase by CD82 has been shown to induce numb phosphorylation at T346S348 and promoted Aβ secretion, which suggests that elevated CD82‐TRPM7‐Numb signal may be associated with cognitive impairment.^43^ Activation of TRPM7 channel prevent AD‐related pathology by increasing basal autophagy and decreasing Aβ secretion.^44^ Therefore, TRPM7 may contribute to AD pathology through regulation of Aβ. SOCS3 has been reported to inhibit cytokine signalling and inflammatory gene expression.^45^ The SOCS3 mRNA level was found significantly altered in AD brain compared with that in non‐demented brain,^46^ hence it may play a role in AD through anti‐inflammatory effect. In addition, SOCS3 may also play a regulatory role in the insulin pathway in AD.^47^

As for OGDH, it constitutes a complex to mediate the decarboxylation of alpha‐ketoglutarate.^48^ Evidence has also shown that the reduction in the activity of the alpha‐ketoglutarate dehydrogenase is associated with AD.^49^, ^50^ Besides, OGDH is also one of the Aβ targets.^51^ These data have indicated that dysregulation of OGDH may also be causation of AD.

However, few studies have been conducted on the link between AD and ATR, CALN1and MOB3A genes. ATR is a serine/threonine protein kinase and checkpoint protein that can be activated by ionizing radiation or ultraviolet radiation (Wright et al., 1998). Jowsey P A etc. found that Ser228 in Fe65 was a novel phosphorylation site that was independently regulated by ATR or ATM.^52^ Based on the alteration of ATR expression and the roles of Fe65 in the Alzheimer's biology and DNA damage response pathway, we have reason to speculate that ATR may be involved in the pathological process of AD. CALN1 encodes a protein with high similarity to the calcium‐binding proteins of the calmodulin family. It negatively regulates Golgi‐to‐plasma membrane trafficking by interacting with PI4KB and inhibiting its activity.^53^ Importantly, CALN1 can participate in the regulation of trans‐synaptic signalling, such as calcium turnover, which plays a role in the physiology of neurons and is potentially important in memory and learning.^54^ MOB3A may modulate kinase activity. However, little is known about its function at present.

Taken together, among these hub genes, TRPM7, SOCS3 and OGDH have been reported that they may contribute to AD, although the evidence is still insufficient and further research is needed to confirm. This also proves the reliability of our approaches for discovery of the hub genes to a certain extent. Whether the CALN1, ATR and OGDH can be real crucial genes in AD remains to be further investigated.

In fact, apart from these hub genes, the candidate miRNAs in the mRNA‐miRNA network may also play a role in AD. It is worth noting that previous studies based on the blood of AD patients showed that they screened out 5 key miRNAs from 853 pairs of mRNA and miRNA interactions by building the network.^29^ Unfortunately, the candidate miRNAs in this study did not overlap with the above 5 miRNAs. This may attribute to the difference in the samples size or in tissues selected in patients.

However, there is a disadvantage in our research. Unlike the construction of the local mRNA‐miRNA network in the prefrontal cortex, it is not reasonable to select mRNA dataset of hippocampus and miRNA dataset of plasma to construct mRNA‐miRNA network. The reason why we do this is no hippocampal miRNA dataset available at present. For the sake of rigour, we have already verified these hub genes through independent datasets and experiments. Nevertheless, influence of samples from different participants and races cannot be ignored. In future, more datasets or experiments are required to confirm these hub genes or miRNAs, and to explore more valuable biomarkers or targets for AD.

## 5 CONCLUSIONS

In this study, six significant hub genes were found by constructing the mRNA and miRNA network. Three of the genes, CALN1, ATR and OGDH are most likely to serve as biomarkers or targets in AD, although further research is needed on these hub genes to explore their specific roles and potential underlying mechanisms in AD, our findings provide potential biomarkers and targets that lead to better prevention and treatment of AD in the future.

## AUTHOR CONTRIBUTIONS

**Zhi‐Hang Huang:** Data curation (lead); resources (lead). **Hai Wang:** Methodology (equal); software (equal). **Dong‐Mei Wang:** Methodology (equal); validation (equal). **Xiu‐Yun Zhao:** Data curation (equal); validation (equal). **Wen‐Wen Liu:** Methodology (equal); software (equal). **Xin Zhong:** Methodology (equal); software (equal). **Dong‐Mei He:** Methodology (equal); software (supporting). **Ben‐Rong Mu:** Project administration (equal); writing – original draft (lead). **Mei‐Hong Lu:** Project administration (equal); writing – review and editing (lead).

## CONFLICT OF INTEREST

The authors declare that there is no conflict of interest regarding the publication of this article.

## Supporting information


**Figure S1** Identification of DEGs in the independent datasets. (A and C) the volcano map showing the DEGs in prefrontal cortex (A) and hippocampus (C). DEGs with |log2 Fold Change| ≥ 0.2 (A) or 1 (C) and *p* value < 0.05 were considered as significantly different. Red and green represent up‐regulated and down‐regulated expression, respectively. (B and D) showing the expression level of all DEGs in each sample. Red and green represent high and low expression respectively

**Table S1** Primers used for qPCR
**TABLE S2** GO and KEGG analysis of 20 candidate hub genes in prefrontal cortex
**TABLE S3** GO and KEGG analysis of 30 candidate hub genes in hippocampus

## Data Availability

The raw data supporting the conclusions of this article will be made available by the authors, without undue reservation.
