# Supplementary figures and images for: Identification of core genes in prefrontal cortex and hippocampus of Alzheimer's disease based on mRNA‐miRNA network
Source: J Cell Mol Med. 2022 Nov 19;26(23):5779–93. doi: 10.1111/jcmm.17593 (PMC9716226; doi:10.1111/jcmm.17593)

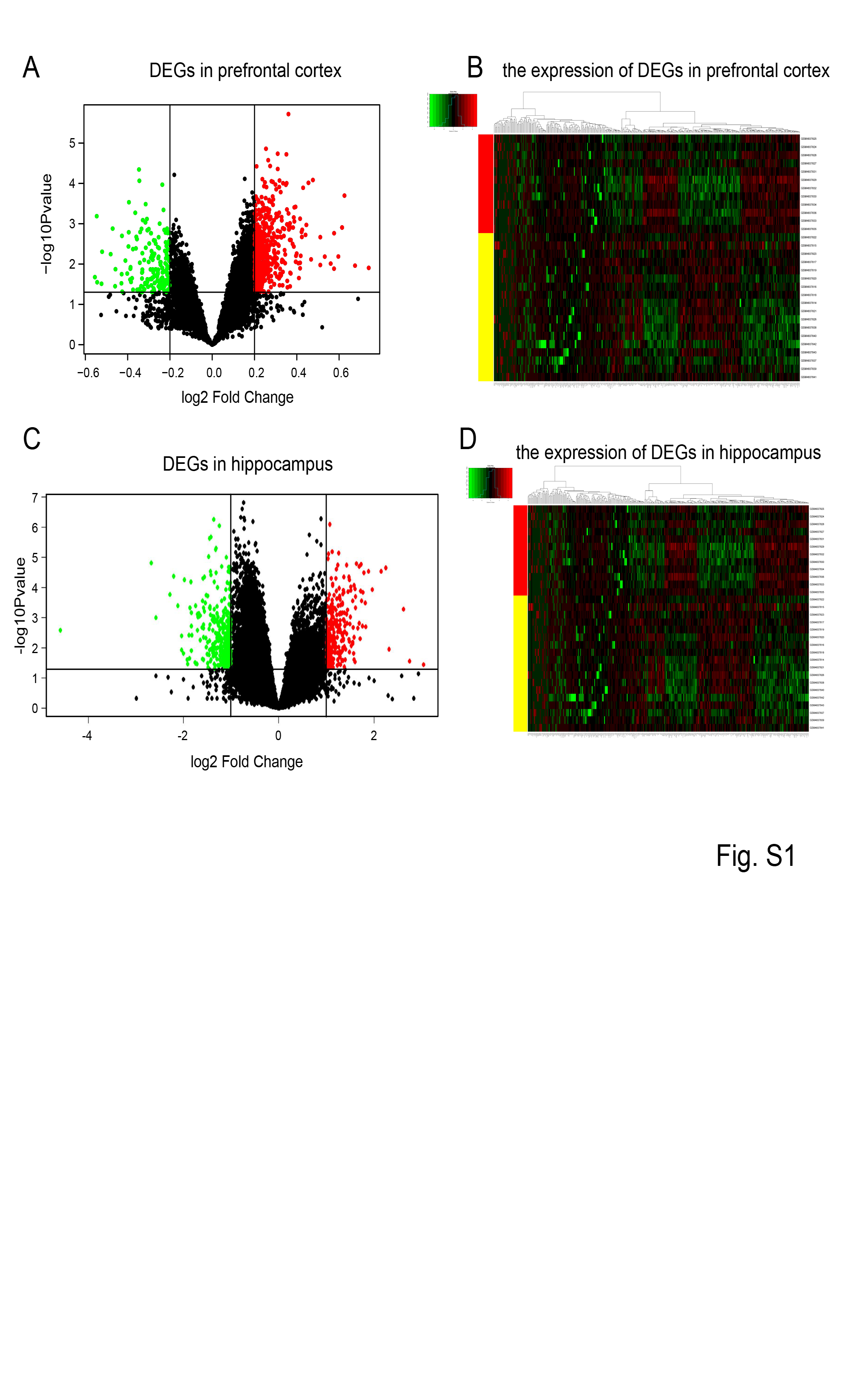

Supplement: Supplementary file 1 — Figure S1 Identification of DEGs in the independent datasets. (A and C) the volcano map showing the DEGs in prefrontal cortex (A) and hippocampus (C). DEGs with |log2 Fold Change| ≥ 0.2 (A) or 1 (C) and p value < 0.05 were considered as significantly different. Red and green represent up‐regulated and down‐regulated expression, respectively. (B and D) showing the expression level of all DEGs in each sample. Red and green represent high and low expression respectively [file JCMM-26-5779-s002.tif]
