# Supplementary material for: Identification of core genes in prefrontal cortex and hippocampus of Alzheimer's disease based on mRNA‐miRNA network
Source: J Cell Mol Med. 2022 Nov 19;26(23):5779–93. doi: 10.1111/jcmm.17593 (PMC9716226; doi:10.1111/jcmm.17593)
Supplement: Supplementary file 2 — Table S1 Primers used for qPCR TABLE S2 GO and KEGG analysis of 20 candidate hub genes in prefrontal cortex TABLE S3 GO and KEGG analysis of 30 candidate hub genes in hippocampus [file JCMM-26-5779-s001.docx]

**Supplementary Tables**

**Table S1. Primers used for qPCR.**

| Gene | Primer pairs (5' - 3') |
| --- | --- |
| TRPM7 | \| Forward \| AGGATGTCAGATTTGTCAGCAAC \| \| --- \| --- \| \| Reverse \| CCTGGTTAAAGTGTTCACCCAA \| |
| \| ATR \| \| --- \| \|  \| | \| Forward \| GAATGGGTGAACAATACTGCTGG \| \| --- \| --- \| \| Reverse \| TTTGGTAGCATACACTGGCGA \| |
| \| SOCS3 \| \| --- \| \|  \| | \| Forward \| ATGGTCACCCACAGCAAGTTT \| \| --- \| --- \| \| Reverse \| TCCAGTAGAATCCGCTCTCCT \| |
| \| PTPN1 \| \| --- \| \|  \| | \| Forward \| GGAACTGGGCGGCTATTTACC \| \| --- \| --- \| \| Reverse \| CAAAAGGGCTGACATCTCGGT \| |
| \| OGDH \| \| --- \| \|  \| | \| Forward \| GTTTCTTCAAACGTGGGGTTCT \| \| --- \| --- \| \| Reverse \| GCATGATTCCAGGGGTCTCAAA \| |
| \| FLCN \| \| --- \| \|  \| | \| Forward \| AACGCCATAGTCGCCCTCT \| \| --- \| --- \| \| Reverse \| CTGCTCATCTGAATGCCACC \| |
| \| MOB3A \| \| --- \| \|  \| | \| Forward \| ACATTCAGGCCCAAACGCAA \| \| --- \| --- \| \| Reverse \| GGCAACCCAGTCGTTGAGA \| |
| \| DDB1 \| \| --- \| \|  \| | \| Forward \| GTGGGAATGTACGGGAAGATTG \| \| --- \| --- \| \| Reverse \| CGCCACTCTGCTTATACTCCAA \| |
| \| EID2 \| \| --- \| \|  \| | \| Forward \| AGCGTCTTGGGCATCAATTAC \| \| --- \| --- \| \| Reverse \| AGGTACTCTATGTCGAAGGCTG \| |
| \| CALN1 \| \| --- \| \|  \| | \| Forward \| ATGCCGTTCCACCATGTAACC \| \| --- \| --- \| \| Reverse \| CACGGAGATATTAGCCAACTGC \| |
| \| GNA13 \| \| --- \| \|  \| | \| Forward \| GTCCAAGGAGATCGACAAATGC \| \| --- \| --- \| \| Reverse \| CCAGCACCCTCATACCTTTGA \| |
| \| APP \| \| --- \| \|  \| | \| Forward \| TCCGAGAGGTGTGCTCTGAA \| \| --- \| --- \| \| Reverse \| CCACATCCGCCGTAAAAGAATG \| |

**Table S2. GO and KEGG analysis of 20 candidate hub genes in prefrontal cortex.**

| Category | Pathway ID | Pathway description | Count | P-value | Genes |
| --- | --- | --- | --- | --- | --- |
| BP | GO:35329 | hippo signaling | 3 | 3.75E-04 | YAP1, WWTR1, STK3 |
|  | GO:45944 | Positive regulation of transcription from RNA polymerase II promoter | 7 | 3.96E-04 | YAP1, WWTR1, CBFB, PPARA, FOXO1, NFE2L2, RUNX1 |
|  | GO:6367 | Transcription initiation from RNA polymerase II promoter | 4 | 5.37E-04 | YAP1, WWTR1, PPARA, CTGF |
|  | GO:30216 | keratinocyte differentiation | 3 | 2.95E-03 | YAP1, TXNIP, EPHA2 |
|  | GO:1649 | osteoblast differentiation | 3 | 5.45E-03 | WWTR1, CBFB, EPHA2 |
| MF | GO:5515 | protein binding | 17 | 3.75E-03 | YAP1, WWTR1, RAB3C, CBFB, TNFRSF10B, FOXO1, STK3, CTGF, RUNX1, GNA13, GNAS, TXNIP, CYCS, PPARA, MCL1, NFE2L2, EPHA2 |
|  | GO:1223 | transcription coactivator binding | 2 | 1.12E-02 | PPARA, FOXO1 |
|  | GO:3714 | transcription corepressor activity | 3 | 2.15E-02 | YAP1, WWTR1, RUNX1 |
|  | GO:4871 | signal transducer activity | 3 | 2.17E-02 | GNA13, UNC13A, GNAS |
|  | GO:31683 | G-protein beta/gamma-subunit complex binding | 2 | 2.23E-2 | GNA13, GNAS |
| CC | GO:5829 | cytosol | 12 | 1.27E-04 | YAP1, WWTR1, RAB3C, DDIT4, GNAS, TXNIP, CYCS, FOXO1, CTGF, STK3, MCL1, NFE2L2 |
|  | GO:5634 | nucleus | 13 | 2.57E-03 | YAP1, WWTR1, CBFB, FOXO1, STK3, RUNX1, GNA13, GNAS, TXNIP, CYCS, PPARA, MCL1, NFE2L2 |
|  | GO:5737 | cytoplasm | 12 | 7.23E-03 | GNA13, YAP1, WWTR1, UNC13A, DDIT4, GNAS, TXNIP, FOXO1, STK3, MCL1, NFE2L2, RUNX1 |
|  | GO:48471 | perinuclear region of cytoplasm | 4 | 2.54E-02 | RAB3C, GNAS, CTGF, CALN1 |
|  | GO:5834 | heterotrimeric G-protein complex | 2 | 3.39E-02 | GNA13, GNAS |

**Table S3. GO and KEGG analysis of 30 candidate hub genes in hippocampus.**

| Category | Pathway ID | Pathway description | Count | P-value | Genes |
| --- | --- | --- | --- | --- | --- |
| BP | GO:0001932 | regulation of protein phosphorylation | 4 | 2.40E-05 | FLCN,SOCS3,SESN2, HUS1 |
|  | GO:0007183 | SMAD protein complex assembly | 3 | 7.46E-05 | SMAD2,EID2,SMAD3 |
|  | GO:0045944 | positive regulation of transcription from RNA polymerase II promoter | 9 | 1.43E-04 | SMAD2,KMT2D,FLCN,  STAT5B,SMARCC1, SMAD3,NCOA3,ARNT,  IKZF3 |
|  | GO:0030512 | negative regulation of transforming growth factor beta receptor signaling pathway | 4 | 1.62E-04 | SMAD2,TGFBR3,EID2,  SMAD3 |
|  | GO:0017015 | regulation of transforming growth factor beta receptor signaling pathway | 3 | 5.00E-04 | SMAD2,EID2,SMAD3 |
| MF | GO:0035326 | enhancer binding | 3 | 2.56E-04 | SMAD2,SMAD3,ARNT |
|  | GO:0005515 | protein binding | 25 | 5.85E-04 | KMT2D,DYRK2,BAZ2A, IKZF3,FLCN,SOCS3,  CD19,SESN2,MAP2K7, SMAD2,PTPN1,STAT5B, SMARCC1,SMAD3, NCOA3,ARNT,HUS1, TGFBR3,DDB1,EID2, MCM5,VIM,IL6ST, RAD18, ATR |
|  | GO:0003677 | DNA binding | 10 | 0.001445 | SMAD2,DDB1,KMT2D, SMARCC1,ZMYM2,  SMAD3,ARNT,BAZ2A,  RAD18,ATR |
|  | GO:0004713 | protein tyrosine kinase activity | 4 | 0.001504 | STAT5B,ZMYM2,DYRK2, MAP2K7 |
|  | GO:0005160 | transforming growth factor beta receptor binding | 3 | 0.002351 | SMAD2,TGFBR3,SMAD3 |
| CC | GO:0005654 | nucleoplasm | 15 | 2.90E-05 | SMAD2,KMT2D, STAT5B,SMARCC1, SMAD3,DYRK2,NCOA3, HUS1,ARNT,BAZ2A,  DDB1,EID2,MCM5,  RAD18,ATR |
|  | GO:0005737 | cytoplasm | 17 | 0.00244 | SMAD2,STAT5B,SMAD3,DYRK2,NCOA3,ARNT,  BAZ2A,IKZF3,TGFBR3,  DDB1,FLCN,SOCS3,  ZMYM2,SESN2,VIM,  MAP2K7,RAD18 |
|  | GO:0071144 | SMAD2-SMAD3 protein complex | 2 | 0.00318 | SMAD2, SMAD3 |
|  | GO:0000790 | nuclear chromatin | 4 | 0.003489 | SMAD2,SMARCC1, SMAD3,NCOA3 |
|  | GO:0005634 | nucleus | 16 | 0.010559 | SMAD2,KMT2D, STAT5B,SMAD3, DYRK2,NCOA3,HUS1, ARNT,BAZ2A,IKZF3, DDB1,FLCN,SESN2, MCM5,MAP2K7,  RAD18 |
| KEGG pathway | hsa04110 | Cell cycle | 4 | 0.004486 | SMAD2,SMAD3,MCM5, ATR |
|  | hsa04520 | Adherens junction | 3 | 0.016036 | SMAD2,PTPN1,SMAD3 |
|  | hsa05166 | HTLV-I infection | 4 | 0.031122 | SMAD2,STAT5B,SMAD3,ATR |
|  | hsa05169 | Epstein-Barr virus infection | 3 | 0.043825 | CD19,VIM,MAP2K7 |
